# Editorial: Nanotoxicology: Challenges and solutions to safeguard human health and the environment

**DOI:** 10.3389/fbioe.2022.1110246

**Published:** 2022-12-07

**Authors:** Ilaria Corsi, Martin Federico Desimone, Jimena Cazenave

**Affiliations:** ^1^ Department of Physical, Earth and Environmental Sciences, University of Siena, Siena, Italy; ^2^ Universidad de Buenos Aires, Consejo Nacional de Investigaciones Científicas y Técnicas (CONICET), Instituto de Química y Metabolismo Del Fármaco (IQUIMEFA), Facultad de Farmacia y Bioquímica, Buenos Aires, Argentina; ^3^ Laboratorio de Ictiología, Instituto Nacional de Limnología (INALI), Consejo Nacional de Investigaciones Científicas y Técnicas (CONICET), Universidad Nacional Del Litoral, Santa Fe, Argentina

**Keywords:** nanotoxicology, safety by design, harmful effects on living organisms including humans, nanoecotoxicology, nanomaterials risk assessment

Undoubtedly, nanotechnology is well established in everyday life as denoted by the great variety of products that contain engineered nanomaterials (ENMs). The release of ENMs and particles to different environmental matrices (water, air, soil) has been demonstrated during the life cycle of consumer products. Consequently, both intentional and unintentional exposure to ENMs have raised concern about their potentially harmful effects on living organisms, including humans, from which emerged the recent research area of nanotoxicology. In this way, different characteristics of the ENMs (i.e., size, shape, charge, chemical composition) have been linked to toxicological effects. Moreover, ENMs can cause toxicity through different mechanisms, ranging from the simple physical adsorption, to the biological surface, to the triggering of complex processes that lead to oxidative stress. Recently, design approaches are gaining importance in the field of nanotechnology with the aim to develop safe and sustainable products.

This Research Topic aims to gather information on the challenges and solutions of the safety by design approach in nanotechnology, with particular emphasis on using nanotoxicology. Therefore, it is intended to present the latest advances to analyze and minimize any toxicological risk to humans and the environment, while maintaining all the properties and characteristics of the ENMs themselves. By bridging the gap between human and environmental nanotoxicology, this Research Topic aims to generate the basis of a reliable discussion of engineered nanomaterial risk assessment along with different biological systems to ensure the safe use of nanotechnology.

This Research Topic presents four original research articles, one perspective article and two reviews.

Toxicity assessments of engineered nanomaterials and nanoparticles (EMN/Ps) using a variety of multispecies organisms have become an essential part of environmental and human safety. In this way, MacCormack et al. evaluated the ecotoxicity of boron oxide nanoparticles (nB_2_O_3_) through several biochemical markers in isolated rainbow trout (*Oncorhynchus mykiss*) hepatocytes and *in vivo* three fish species with different sensitivities. The main results showed that nB_2_O_3_ effects on metabolism, ionoregulation and neurotransmission were species-specific. However, toxic effects were provoked at nB_2_O_3_ concentrations which exceed those proposed as environmentally relevant. Overall, nano-specific effects of nB_2_O_3_ were minor and similar to their bulk material counterpart (boric acid); so, it could represent a promising formulation for further developing safe and sustainable nanoproducts.

Another study of this Research Topic focused on an enginereed nanomaterial widely used in consumer products, silica nanoparticles (SiNP). For a better understanding of the impact of SiNP on human safety, Almanaa et al. assessed multiple responses in male rats after oral administration of SiNP. The results revealed behavioural changes, hepatotoxicity, nephrotoxicity, and immunotoxicity induced by SiNP, which evidence impaired physiological status. Information from this study can be useful for further recommendations regarding safe therapeutic doses and also safety measures to decrease hazards to the environment.

In the review manuscript by Mortimer et al. the advantages in using omics tools in assessing how bacteria respond to engineered nanomaterials across three physiological effect thresholds as inhibitory, sub-inhibitory or stimulatory are critically discussed. In comparison with classical endpoints as population growth or viability, omics provide clues on the ability of ENMs to affect a variety of bacterial cellular pathways and allow recognition of concentration-dependent trends. Such knowledge can be used to tune application designs in different areas from nanomedicine to nano-enabled agrochemicals.

The contribution by Perucca et al. the need for promoting a Sustainable and Safe-by-Design (SSbD) holistic approach for ENMs which allow to achieve human and environmental protection, support industrial relevance, societal empowerment and regulatory preparedness is discussed. In such context, a data driven Management Methodology developed in the framework of the ASINA project (ASINA SMM) capturing quality, safety and sustainability criteria across the Nano-Enabled Products (NEPs) life-cycle is presented. Three main pillars are identified as environmental impact, technoeconomic performance, functionality and human and environmental safety, all jointly aiming to achieve SSbD development of ENMs.

The work developed by Peng et al., provides an insightful panorama of the mechanisms involved in the toxic effect of fine particles (PM_2.5_). The authors clearly identified candidate genes associated with PM_2.5_ toxicity. Indeed, downregulating these genes increased cell viability and attenuated apoptosis in cells exposed to PM_2.5_. It is worth to mention that PM_2.5_ carries metals (Zn, Co, Cd) which can pass through the alveolar epithelium and enter the circulatory system and tissues. In this sense, it is claimed that the identified genes may be contributing to the absorption of metals and consequently induce apoptosis mediated by ROS.

Muhammad et al., provides an insightful alternative to mitigate the chronic lead toxicity. In this sense, the *in vitro* and *in vivo* bioremediation potentials of orally supplemented free and microencapsulated *Lactobacillus acidophilus* KLDS strains were evaluated. Interestingly, orally administered free and microencapsulated KLDS provided significant protection by reducing Pb levels in blood, kidney and liver. The authors conclude that free and microencapsulated *L. acidophilus* KLDS could be considered as true candidates to alleviate chronic Pb toxicity.

Finally, the article by Corsi et al., summarizes and integrates the current state of knowledge on the critical characteristics of silver nanoparticles (AgNPs) that need to be addressed in the safe by design process to reduce any potential toxicological risks associated with their exposure to living organism. Particularly, the authors review the ecotoxicological effects documented on freshwater and marine species that demonstrate the importance of the relationship between the nanoparticle design and their biological outcomes in terms of environmental safety.

